# Photosensitization and mechanism of cytotoxicity induced by the use of ALA derivatives in photodynamic therapy

**DOI:** 10.1054/bjoc.2001.1875

**Published:** 2001-07

**Authors:** A Casas, H Fukuda, G Di Venosa, A Batlle

**Affiliations:** Centro de Investigaciones sobre Porfirinas y Porfirias (CIPYP) FCEyN (University of Buenos Aires and CONICET); Ciudad Universitaria, Pabellón II, 2do piso; (1428) Capital Federal, Argentina

**Keywords:** photodynamic therapy, PDT, aminolevulinic acid, ALA, ALA derivatives, apoptosis

## Abstract

The use of more lipophilic derivatives of 5-aminolevulinic acid (ALA) is expected to have better diffusing properties, and after conversion into the parent ALA, to reach a higher protoporphyrin IX (PPIX) formation rate, thus improving the efficacy of topical photodynamic therapy (PDT). Here we have analysed the behaviour of 3 ALA derivatives (ALA methyl-ester, hexyl ester and a 2-sided derivative) regarding PPIX formation, efficiency in photosensitizing cells and mechanism of cellular death. The maximum amount of porphyrins synthesized from 0.6 mM ALA was 47 ± 8 ng/10^5^ cells. The same amount was formed by a concentration 60-fold lower of hexyl-ALA and 2-fold higher of methyl-ALA. The 2-sided derivative failed to produce PPIX accumulation. Applying a 0.6 J cm^−2^ light dose, cell viability decreased to 50%. With the 1.5 J cm^−2^ light dose, less than 20% of the cells survive, and higher light doses produced nearly total cell killing. Comparing the PPIX production and the induced phototoxicity, the more the amount of porphyrins, the greater the cellular killing, and PPIX formed from either ALA or ALA-esters equally sensitize the cells to photoinactivation. ALA-PDT treated cells exhibited features of apoptosis, independently on the pro-photosensitizer employed. ALA-PDT can be improved with the use of ALA derivatives, reducing the amount of ALA necessary to induce efficient photosensitization. ©2001 Cancer Research Campaign http://www.bjcancer.com


*British Journal of Cancer* (2001) **85**(2), 279–284
© 2001 Cancer Research Campaign
doi: 10.1054/ bjoc.2001.1875, available online at http://www.idealibrary.com on IDEAL®

http://www.bjcancer.com

### Photosensitization and mechanism of cytotoxicity induced by the use of ALA derivatives in photodynamic therapy

**A Casas, H Fukuda, G Di Venosa and A Batlle**

Centro de Investigaciones sobre Porfirinas y Porfirias (CIPYP) FCEyN (University of Buenos Aires and CONICET); Ciudad Universitaria, Pabellón II, 2do piso; (1428) Capital Federal, Argentina

**Summary** The use of more lipophilic derivatives of 5-aminolevulinic acid (ALA) is expected to have better diffusing properties, and after conversion into the parent ALA, to reach a higher protoporphyrin IX (PPIX) formation rate, thus improving the efficacy of topical photodynamic therapy (PDT). Here we have analysed the behaviour of 3 ALA derivatives (ALA methyl-ester, hexyl ester and a 2-sided derivative) regarding PPIX formation, efficiency in photosensitizing cells and mechanism of cellular death. The maximum amount of porphyrins synthesized from 0.6 mM ALA was 47 ± 8 ng/$10^5$ cells. The same amount was formed by a concentration 60-fold lower of hexyl-ALA and 2-fold higher of methyl-ALA. The 2-sided derivative failed to produce PPIX accumulation. Applying a 0.6 J $cm^{-2}$ light dose, cell viability decreased to 50%. With the 1.5 J $cm^{-2}$ light dose, less than 20% of the cells survive, and higher light doses produced nearly total cell killing. Comparing the PPIX production and the induced phototoxicity, the more the amount of porphyrins, the greater the cellular killing, and PPIX formed from either ALA or ALA-esters equally sensitize the cells to photoinactivation. ALA-PDT treated cells exhibited features of apoptosis, independently on the pro-photosensitizer employed. ALA-PDT can be improved with the use of ALA derivatives, reducing the amount of ALA necessary to induce efficient photosensitization. © 2001 Cancer Research Campaign http://www.bjcancer.com

**Keywords**: photodynamic therapy; PDT; aminolevulinic acid; ALA; ALA derivatives; apoptosis

In recent years, 5-aminolevulinic acid (ALA)-mediated photodynamic therapy (PDT) has become one of the most promising field in PDT research. ALA is the pro-drug of the photosensitizer protoporphyrin IX (PPIX). After ALA administration, cells generate PPIX through the haem biosynthetic pathway. The main advantage of PPIX relative to other photosensitizers is the short half-life of its photosensitizing effects, which do not last longer than 48 h (Kennedy et al, 1990; Fukuda et al, 1993). Besides, ALA-induced porphyrin fluorescence may also assist in the early detection of some malignancies (Kriegmair et al, 1996).

ALA-based PDT is also showing promise in the treatment of other dermatological disorders such as nonmelanoma skin cancer, patch and plaque stage cutaneous T-cell lymphoma, psoriasis and alopecia areata (Lui and Anderson, 1992; Oseroff, 1993). In addition, other cancer types such as lung (Baumgartner et al, 1996), bladder (Jichlinski et al, 1997), oral cavity (Fan et al, 1996), oesophagus (Gossner et al, 1998), endometrious (Wyss et al, 1998) and brain (Stummer et al, 1998) have been recently treated with ALA-PDT.

The hydrophilic nature of the ALA molecule limits somehow the penetration through the straum corneum of the skin. Hence, ALA-induced PPIX formation is often restricted to superficial tissue layers because of both inhomogeneous and partial tissue distribution in deeper-lying or nodular lessions. Different approaches are currently under investigation to enhance penetration, such as the application of ALA in various vehicles and the development of new synthetic molecules derived from ALA. The importance of the vehicle formulation in topical ALA-PDT has been extensively studied by Casas et al (2000).

The use of more lipophilic derivatives of ALA was expected to have better diffusing properties, and after conversion into the parent ALA by enzymatic hydrolysis, to reach a higher PPIX formation rate.

Kloek et al (1996, 1998) synthesized a range of ALA esters using alcohols of increasing carbon chain length, and demonstrated in cell lines and animal models, that several ALA pro-drugs are capable of being taken up, desterified and converted into PPIX with higher efficiency than ALA itself. Gaullier et al (1997) found that long-chained ALA esters reduced 30–150-fold the amount of ALA needed to reach the same level of PPIX accumulation as that obtained with non-esterified ALA in a human cell line, whereas short-chained pro-ALAs were less efficient than ALA.

Some esterified ALA derivatives have been used in normal mouse skin (Peng et al, 1996), in human basal cell carcinomas (Peng et al, 1995) and in human bladder tumours (Lange et al, 1996) and a higher and more homogeneous tissue distribution was produced when compared to that of free ALA-induced porphyrins.

Regarding the subcellular target of PDT damage induced by photodynamic treatment, it is well known that singlet oxygen and other reactive oxygen species lead to lipid peroxidation and damage to biological membranes, DNA, cytoskeleton, etc. (Moan and Berg, 1992). But the final mechanism of cell death may be either necrosis or apoptosis, depending on the photosensitizer, concentration and time of exposure, light dose, light source and cell type (He et al, 1994; Dellinger, 1996; Noodt et al, 1996; Miyamoto et al, 1999). Activation of phospholipases (Agarwall et al, 1993) and caspases (He et al, 1998) have been shown to be involved in the PDT-induced apoptotic process.

*Received 7 September 2000*
*Revised 26 March 2001*
*Accepted 29 March 2001*

*Correspondence to:* A Batlle, Viamonte 1881 10A, 1056 Buenos Aires, Argentina. E-mail: batlle@mail.retina.ar

Webber et al (1996) reported an apoptotic response to PDT performed in vivo in a murine colon adenocarcinoma with endogenously formed PPIX from ALA. Noodt et al (1996) observed that some cell lines exposed to ALA-PDT die mainly by necrosis, whereas others die by apoptosis, yet there have been no reports on the type of cytotoxicity induced by ALA-pro-drugs.

Here we have analysed the behaviour of 3 ALA derivatives (ALA methly-ester, hexyl ester and a 2-sided derivative) that had been previously tested in rat and human skin explants (Casas et al, 1999) regarding PPIX formation and their efficiency in sensitizing cells to photoinactivation. The mechanism of cellular death induced by ALA and ALA derivatives is also discussed.

### MATERIALS AND METHODS

### Cell line and cell culture

Cell line LM2 (Galli et al, 2000) derived from the murine mammary adenocarcinoma M2 was cultured in minimum essential Eagle's medium (MEM), supplemented with 2 mM L-glutamine, 40 μg gentamycin $ml^{-1}$ and 5% fetal bovine serum (FBS), and incubated at 37°C in an atmosphere containing 5% $CO_2$.

### Chemicals

ALA and ALA-methyl-ester (Me-ALA) were obtained from Sigma Chem Co; ALA-Hexyl ester (He-ALA) and Carbobenzoyloxy-D-phenyl-alanyl-5-ALA-ethyl ester (CDF-ALA) were synthesized according to the procedures described in Casas et al (1999).

ALA, Me-ALA and He-ALA were dissolved in saline. CDF-ALA was dissolved in DMSO in a concentration such that 0.6 mM CDF-ALA corresponds to 0.66 mM DMSO. ALA and ALA derivatives solutions were sterilized by filtration through 0.21 μm pore size filters. Addition of either ALA or ALA derivatives to cells did not change the pH of the medium.

### Laser irradiations

Cells were incubated in serum-free medium containing ALA or ALA derivatives, and 3 h later, laser irradiations were performed. After 5 minutes of irradiation, medium was replaced by ALA-free medium + FBS, the cells were incubated for another 19 h and then tested for viability. Irradiations were performed employing a rhodamine dye laser (Model DL30, Oxford Lasers) pumped by a copper vapour laser (CU15A, Oxford Lasers) tuned to 630 nm. The light was focused into a 400-μm-diameter optical fibre coupled to a frontal light distributor (Model FD2, Medlight, Ecublens, Switzerland) to produce a treatment area of uniform intensity. The output power from the fibre was measured with a power meter (Model LM-100XL, Coherent, Auburn, CA) before each application, and adjusted to the desired light dose.

### MTT viability assay

Phototoxicity/toxicity was documented by the MTT assay (Denizot and lang, 1986). Following appropriate treatments, MTT (3-[4,5-dimethylthiazol-2-yl]-2,5-diphenyltetrazoliumbromide) solution was added to each well in a concentration of 0.5 mg $ml^{-1}$, and plates were incubated at 37°C for 1 h. The resulting formazan crystals were dissolved by the addition of DMSO and absorbance was read at 560 nm.

### Measurement of porphyrin synthesis

Cells were exposed to ALA or ALA derivatives in serum-free medium. After incubation, porphyrins accumulated within the cells were extracted twice with 5% HCl, leaving the cells for half an hour in the presence of the acid. These conditions proved to be the optima for total PPIX extraction. The media were acidified and measured directly fluorometrically. The excitation and emission wavelengths of light used producing the highest fluorescence were 406 nm and 604 nm respectively. PpIX (Porphyrin Products, Logan, Utah, USA) was used as a reference standard.

### Cell number

The number of cells seeded per well and the cell number employed for the calculations of porphyrins per cell were determined by counting viable cells with the Trypan blue exclusion method.

### Analysis of DNA fragmentation

The cells treated with PDT, attached and unattached, were harvested 19 h after treatment. The pellets were washed in phosphate-buffered saline, resuspended in TNE (10 mM Tris-HCl, pH 7.6; 1 mM EDTA; and 140 mM NaCl) and lysed at 37°C in 1 ml of extraction buffer (10 mM Tris-HCl, pH 8.0; 100 mM EDTA, pH 8.0; 20 μg $ml^{-1}$ pancreatic RNAse and 0.5% SDS). After 2 h, proteinase K was added at a final concentration of 100 μg $ml^{-1}$ and the mixture was incubated for another 3 h at 50°C. The DNA was extracted twice with equal volumes of phenol and once with chloroform. The DNA was then precipitated with 0.1 vol of sodium acetate (pH 4.8) and 2.5 vol of ethanol at –20°C overnight and pelleted at 14.000 ***g*** for 30 min. Samples were electrophoresed in a 2% agarose gel for approximately 2.5 h at 100 V and DNA was visualized by ethidium bromide staining.

### Morphological studies

The cells were seeded in cover slips, treated with PDT as indicated above, and after 19 h they were stained with acridine orange (5 μg $ml^{-1}$). The samples were washed with MEM, then the cover slips inverted on a slide and fluorescence was observed exciting the sample at 450–490 nm and employing the 510 nm dichroic mirror and 520 nm high-pass filter.

### Statistical treatment

The values are expressed in the figures as mean ± standard error of the mean and they are the average of 3 independent experiments run in triplicate.

### RESULTS

### Cell, time and ALA concentration dependence

Maximal porphyrin synthesis with 0.6 mM ALA-and 3 h incubation was observed between 3 and $3.5 \times 10^4$ cells. When the cell number was increased above $3.5 \times 10^4$, a slight decrease in the tetrapyrrole synthesis per number of cells was found, and employing a cell number below $3 \times 10^4$ cells, accumulation of porphyrins was very low.

© 2001 Cancer Research Campaign

The kinetics of intracellular PPIX accumulation is depicted in Figure 1. It was found that cellular porphyrin content increased linearly during that period of time, indicating that no saturation of enzymatic functions occurred. Similar profiles were obtained using higher ALA concentrations.

In Figure 2 we can observe that the rate of PPIX synthesized increased with ALA or pro-ALA concentration, showing a sigmoidal-profile on logaritmic scales, in the case of ALA and ALA esters. CDF-ALA exhibited a peak at 0.2 mM, and then porphyrins decreased to basal levels.

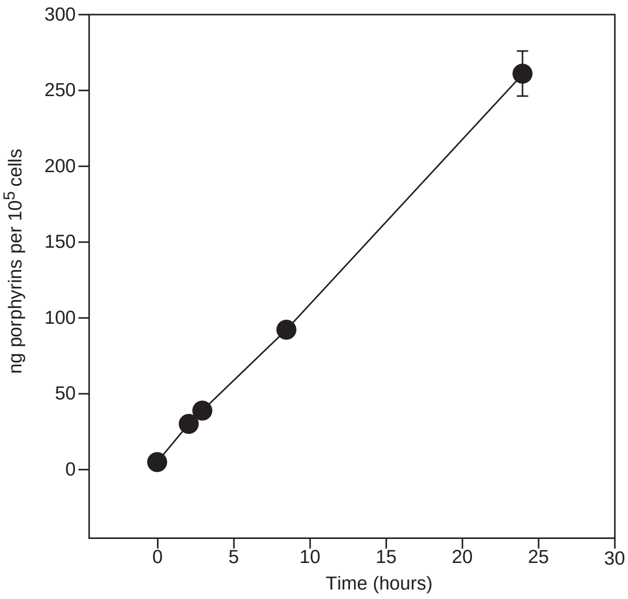

**Figure 1** Porphyrin synthesis as a function of incubation time in the presence of ALA. $3.5 \times 10^4$ cells per well were incubated during different time periods in the presence of 0.6 mM ALA in 24-well plates. Intracellular porphyrins were determined fluorimetrically and relativized per number of cells present at the beginning of the experiment

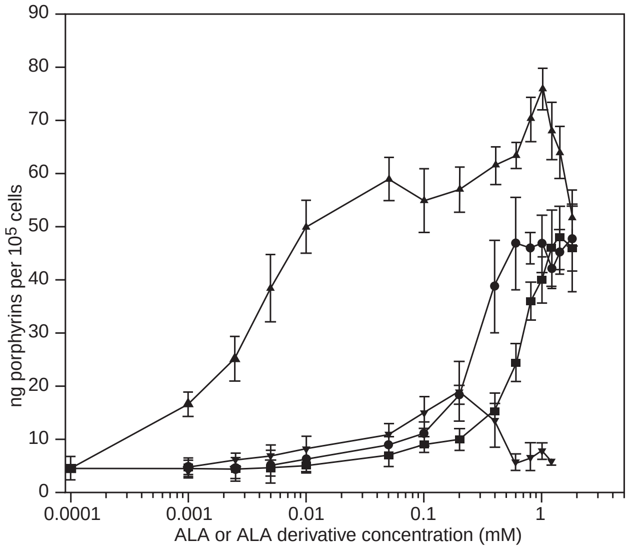

**Figure 2** Porphyrin synthesis from ALA and ALA derivatives. $3.5 \times 10^4$ cells per well were incubated for 3 hours in the presence of different amounts of ALA (●) or its derivatives Me-ALA (■), He-ALA (▲) or CDF-ALA (▼). All compounds were dissolved in water, except for CDF-ALA, which was dissolved in DMSO. Intracellular porphyrins were determined fluorimetrically and relativized per number of cells present at the beginning of the experiment

The maximum amount of porphyrins synthesized from 0.6 mM ALA was 47 ± 8 ng $10^{-5}$. The same amount was formed by a concentration of He-ALA 60-fold lower (0.01 mM). Instead, 2-fold higher Me-ALA (1.2 mM) was needed to reach PPIX biosynthesis equal to that formed from 0.6 mM ALA.

The highest PPIX biosynthesis obtained (expressed as ng $10^{-5}$ cells) was 75 ± 4 from 1 mM He-ALA, 47 ± 8 from 0.6 mM ALA, 48 ± 6 from 1.4 mM Me-ALA and 20 ± 5 from 0.2 mM CDF-ALA. Increasing these concentrations, PPIX accumulation became lower for He-ALA and CDF-ALA. Instead, ALA and Me-ALA maintained a plateau after reaching their maximal level of porphyrins.

Porphyrins released to the media were about 10% of the amount retained intracellularly at all the concentrations of ALA and ALA esters assayed (data not shown). However, 50% of porphyrin release was observed when CDF-ALA was employed at concentrations above 0.6 mM.

In Figure 3 we can see that ALA and Me-ALA are not toxic for the cells. On the other hand, He-ALA at concentrations higher than

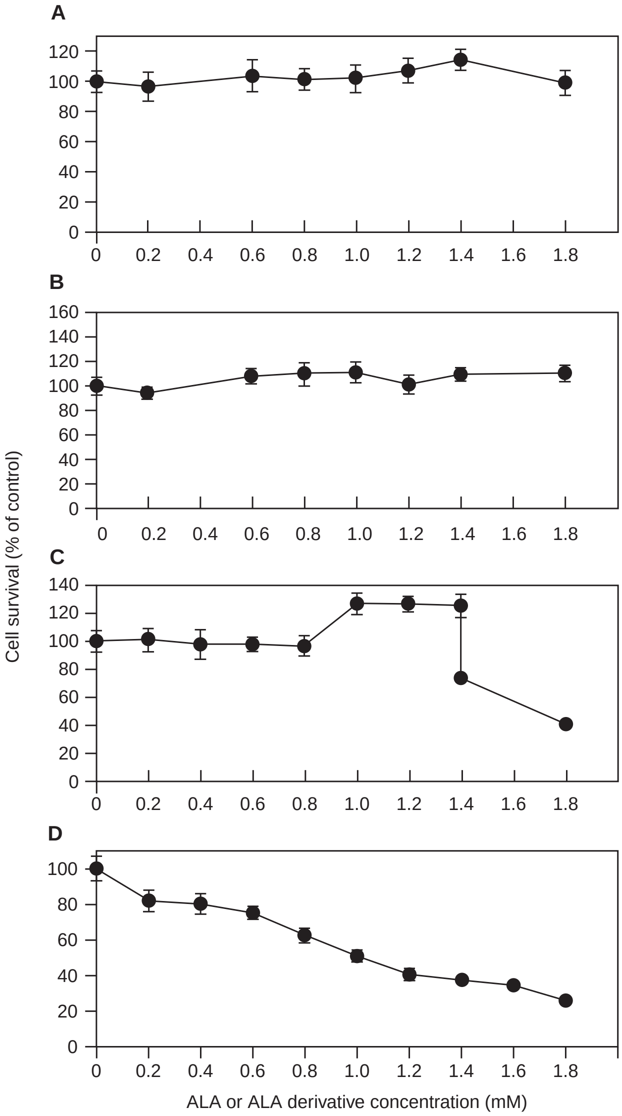

**Figure 3** Dark toxicity of ALA and ALA derivatives. $3.5 \times 10^4$ cells per well were incubated for 3 hours in the presence of different amounts of ALA (**A**), Me-ALA (**B**), He-ALA (**C**) and CDF-ALA (**D**). Immediately after, the MTT assay was performed. Cell survival was expressed as percentage of the control not exposed to ALA

**Table 1** Cell survival after PDT with different light doses

| | ALA | He-ALA | Me-ALA |
|---|---|---|---|
| Control | 100 ± 7 | 100 ± 7 | 100 ± 7 |
| 0.6 J cm$^{-2}$ | 45 ± 5.6 | 60 ± 8.2 | 55 ± 4.8 |
| 1.5 J cm$^{-2}$ | 18 ± 2.4 | 25 ± 3.1 | 27 ± 3.2 |
| 3.0 J cm$^{-2}$ | 4.1 ± 0.9 | 2.5 ± 0.9 | 2.0 ± 0.3 |
| 15 J cm$^{-2}$ | 3.9 ± 0.2 | 3.2 ± 0.6 | 2.5 ± 0.4 |
| 18 J cm$^{-2}$ | 2.8 ± 0.2 | 2.3 ± 0.1 | 3.0 ± 2.2 |

PDT experiments were performed in 6 wells plates. Cells were incubated with 0.6 mM ALA, 0.01 mM He-ALA or 1.2 mM Me-ALA (concentrations producing similar porphyrin accumulation) in FBS-free medium during 3 h. Immediately after, cells were irradiated with different light doses and incubated in medium containing FBS for further 19 h. MTT assay was performed and cell survival were expressed as percentage of the non-irradiated control incubated in the presence of ALA or ALA derivative.

**Table 2** Cell survival after PDT with different ALA or ALA derivative concentrations

| | ALA | He-ALA | Me-ALA |
|---|---|---|---|
| Control | 100 ± 7 | 100 ± 7 | 100 ± 7 |
| 0.005 mM | 100 ± 8 | 54.9 ± 6 | 100 ± 9 |
| 0.01 mM | 100 ± 8 | 1.3 ± 0.3 | 100 ± 7 |
| 0.05 mM | 100 ± 5 | 2.5 ± 0.5 | 100 ± 7 |
| 0.1 mM | 95 ± 5 | 1.2 ± 0.7 | 100 ± 6 |
| 0.2 mM | 80 ± 3 | 1.3 ± 0.4 | 90 ± 5 |
| 0.3 mM | 50 ± 4 | 1.5 ± 0.25 | 85 ± 4 |

PDT experiments were performed in 6 wells plates. Cells were incubated with different concentrations of ALA, He-ALA or Me-ALA in FBS-free medium during 3 h. Immediately after, cells were irradiated with 9 J cm$^{-2}$ and incubated in medium containing FBS for further 19 h. MTT assay was performed and survival percentages were referred to the non-irradiated control incubated in the presence of ALA or ALA derivative.

1.4 mM appeared to be cytotoxic in the dark. CDF-ALA induced a concentration-dependent decrease on cell viability; with 1.2 mM CDF-ALA, less than 40% of cells survive. This effect correlates well with toxicity driven by the DMSO vehicle alone (data not depicted). However, DMSO at concentrations lower than 5% (corresponding to 0.6 mM CDF-ALA) exerted no cytotoxicity measured by the MTT assay.

### PDT-induced cytotoxicity

Survival of irradiated cells exposed to similar porphyrin concentration proceeding form ALA or ALA derivatives is shown in Table 1. When 0.6 J cm$^{-2}$ light dose was used, cell viability decreased approximately to 50% employing all ALA compounds. With the 1.5 J cm$^{-2}$ light dose, less than 20% of the cells survive. Higher light doses produced almost total cell killing. No significant differences between ALA and pro-ALAs were observed for each light dose.

Table 2 depicts the efficacy of PDT with increasing concentrations of ALA and ALA derivatives. We can observe that using a concentration as low as 0.005 mM of He-ALA, cell viability decreases almost to 50%. Increasing the concentration of the derivative to 0.01 mM, cell viability is further reduced, and only 1–3% of cells survive. To reach a similar rate of cell death employing ALA, the concentration must be nearly 60 times higher (0.3 mM ALA induces 50% of cell death). Moreover, when Me-ALA was tested, we found that 0.3 mM of this derivative is necessary to produce 80% of cell death.

### Evidence of apoptosis

Gel electrophoresis was used to detect the appearance of nucleosome multimers after PDT treatment with ALA or ALA derivatives (Figure 4). There was no evidence of internucleosomal cleavage in non-treated cells or in cells treated with ALA, ALA derivatives or light alone, and these lanes contained only high molecular weight DNA. A small amount of necrosis-induced random cut DNA of low molecular weight was detected in the lanes of ALA or ALA derivative controls not exposed to light. When cells were exposed to even low ALA or ALA derivatives concentrations and to a rather high light dose (3 J cm$^{-2}$) (Figure 4A), we detected the typical ladder pattern of nucleosome multimers, yielding 180-base pair integer fragments. In all PDT-treated cells, a background of intranucleosomal cut DNA produced either by necrosis and/or post-apoptotic necrosis processes was observed. Applying light doses higher than 3 J cm$^{-2}$, similar patterns were obtained (data not shown). On the other hand, exposing cells to half the light dose (1.5 J cm$^{-2}$) no detectable evidence of nucleosome-sized fragments was found, independently on the ALA or ALA derivative dose employed (Figure 4B). Intranucleosomal random-cut DNA was also observed in all lanes.

PDT-induced apoptosis was also investigated at the morphological level by staining cells with acridine orange (Figure 5). Normal chromatin was found in control cells, but condensed and fragmented nuclei and condensed cytoplasms were seen after PDT with ALA or ALA derivatives. In addition, few cells can be

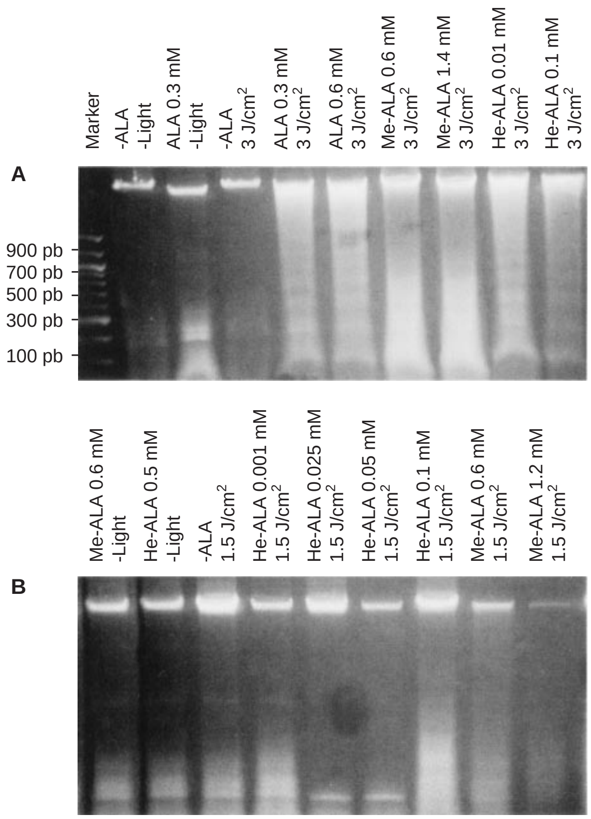

**Figure 4** Agarose gel electrophoresis of DNA from PDT-treated cells. Cells were exposed 3 h to different concentrations of ALA or ALA derivatives and irradiated with 3 J cm$^{-2}$ (**A**) or 1.5 J cm$^{-2}$ (**B**). Non-ALA treated and non-irradiated controls were included. After 19 h DNA was extracted and electrophoresed. The gels were stained with ethidium bromide and visualized under 312 nm light

*© 2001 Cancer Research Campaign*

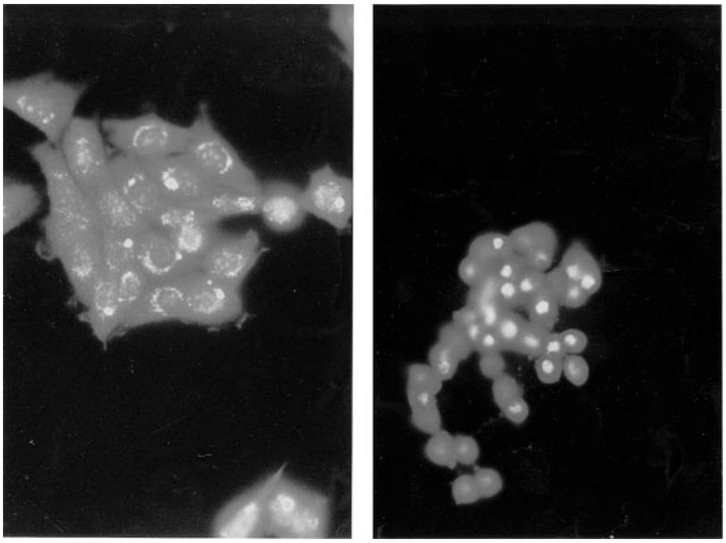

**Figure 5** Fluorescence microscopy showing apoptotic cells. Cells were stained with acridine orange, 19 hours after photodynamic treatment with 0.1 mM He-ALA and a light dose of 3 J cm$^{-2}$. Similar images were obtained with ALA and Me-ALA. Control cells: non-ALA, non-light treated observed 19 h after PDT treatment due to detachment of death cells. Increasing ALA or ALA-derivative doses and light doses, a greater number of apoptotic cells were observed. ALA alone or light alone-treated cells also presented a normal diffusely stained chromatin.

### DISCUSSION

Our results show that the production of PPIX from exogenously added ALA is cell density-dependent, in agreement with other author's reports (Steinbach et al, 1995; Gaullier et al, 1997). Using human lung fibroblasts and colon adenocarcionoma cells, Moan et al (1998) found a similar pattern to ours, and the number of cells per area found to produce maximal porphyrin biosynthesis was also within the same order as that reported here.

The same amount of PPIX is formed by a concentration 60-fold lower of He-ALA and 2-fold higher of Me-ALA than the ALA concentration inducing maximal accumulation of porphyrins. Similar results were observed by other authors with different cell lines (Gaullier et al, 1997; Uehlinger et al, 2000). At their optimal concentrations, ALA and Me-ALA formed an equal amount of porphyrins while PPIX levels from He-ALA were 1.6 times higher. However, we want to point out that the amount of PPIX necessary to produce total cell killing in this model is much lower than those reached under these optimal conditions.

ALA and Me-ALA maintained a plateau after reaching their maximal level of porphyrins. On the contrary, accumulation became lower at high concentrations of He-ALA and CDF-ALA, which corresponded well with dark toxic effects. In addition, the higher release of PPIX when using CDF-ALA more than 0.2 mM, may be ascribed to cell permeability changes driven by high concentrations of the vehicle DMSO (Yyu and Quinn, 1998). In a parallel set of experiments employing ALA and DMSO, we confirmed that, in cells exposed to ALA, DMSO induced the same rate of tetrapyrrole liberation (data not shown).

In agreement with Washbrook and Riley (1997) and Kloek et al (1998) findings, we showed that Me-ALA can not generate PPIX amounts higher than those resulting from ALA. However, Fritsch et al (1998) found that the ratio of porphyrins in solar keratoses versus adjacent normal skin was higher when using this ester. So, in spite of a lower efficiency of Me-ALA as a pro-photosensitizer, better selectivity could result in an advantage which may be exploited for the treatment of certain malignancies.

All ALA compounds produced a plateau value of porphyrins at different concentrations. In this regard, 2 processes: uptake and ester cleavage are necessary for PPIX formation from ALA esters. The facts that different carrier proteins appear to be involved in the transport of ALA and ALA esters (Rud et al, 1999) and that some specific esterases may be involved in the clevage of the different ALA derivatives may be related to their dose-dependent efficacy as pro-photosensitizers.

Comparing the PPIX production with the induced phototoxicity, we found that the more the porphyrin production, the greater the cellular killing. Keeping in line with the findings of Gaullier et al (1997) employing both human and animal cell cultures, we observed here that PPIX formed from ALA and ALA-esters equally sensitize the cells to photoinactivation.

ALA-PDT treated cells exposed to rather high light doses, exhibited morphological features typical of apoptosis, accompanied by the characteristic internucleosomal DNA fragmentation, independently on the pro-photosensitizer employed. These findings are indicating that, after conversion into porphyrins, the remaining ALA-esters or released alcohol chains do not interfere with the mechanism of cytotoxicity induced by photosensitization. The presence of a background smear of DNA, product of random DNA digestion, in all cells PDT-treated with ALA or ALA esters, suggests that necrosis is also induced, although we did not quantified the proportion of cells dying by either necrosis or apoptosis. Traces of random-cut DNA present in cells exposed to ALA or ALA derivatives, suggest that some necrosis is induced by

*© 2001 Cancer Research Campaign*

dark toxicity of these compounds, in spite that no significant loss of viability was detected in the MTT assay at these concentrations.

Reducing the ALA dose required to induce a photosensitizing effect would result in less side effects. Moreover, because the photosensitizing efficacy of ALA-PDT depends on both the amount of PPIX accumulated and the light dose applied, the fluence rate can be substantially decreased if a higher accumulation of porphyrins is reached by employing ALA derivatives, therefore improving ALA-PDT.

### ACKNOWLEDGEMENTS

This research was supported by grants from the Argentine National Research Council (CONICET) (PIP 4108/96 and 105508/98–99), the Science and Technology Argentine Agency (STAA) (PICT 05–00000–01861) and the Association for International Cancer Research (AICR-UK, 98–6). The authors are very grateful to Mrs Victoria Castillo for her skillful technical assistance, and to Dr Elisa Bal de Kier Joffé for her help with fluorescence micrographs. AM del CB and HF hold the posts of Superior and Associate Researchers at the CONICET. The financial support of Ministerio de Salud de la Nación Argentina is also acknowledged.

### REFERENCES

Agarwall M, Larkin H, Zaidi S, Mukhtar H and Oleinick N (1993) Phospholipase activation triggers apoptosis in photosensitized mouse lymphoma cells. *Cancer Res* **53**: 5897–5902

Baumgartner R, Huber R, Schulz H, Stepp H, Rick K, Gamarra F, Leberig A and Roth C (1996) Inhalation of 5-aminolevulinic acid: a new technique for fluorescence detection of early stage lung cancer. *J Photochem Photobiol B* **36**: 167–174

Casas A, Batlle A, Butler A, Robertson D, Brown E, MacRobert A and Riley P (1999) Comparative effect of ALA derivatives on Protoporphyrin IX production in human and rat skin organ cultures. *Br J Cancer* **80**: 1525–1532

Casas A, Fukuda H, Di Venosa G and Batlle A (2000) The influence of the vehicle on the synthesis of porphyrins after topical application of ALA. Implications in cutaneous photodynamic sensitization. *Br J Dermatol* **143**: 1–10

Dellinger M (1996) Apoptosis or necrosis following Photofrin photosensitization: influence of the incubation protocol. *Photochem Photobiol* **64**: 182–187

Denizot F and Lang R (1986) Rapid colorimetric assay for cell growth and survival. Modifications to the tetrazolium dye procedure giving improved sensitivity and reliability, *J Immunol Methods* **89**: 271–277

Fan K, Hopper C, Speight P, Buonaccorsi G, Macrobert A and Bown S (1996) Photodynamic therapy using 5-aminolevulinic acid for premalignant and malignant lesions of the oral cavity. *Cancer* **78**: 1374–1383

Fritsch C, Homey B, Stahl W, Lehmann P, Ruzicka T and Sies H (1998) Preferential relative porphyrin enrichment in solar keratoses upon topical application of delta-aminolevulinic acid methylester. *Photochem Photobiol* **68**: 218–221

Fukuda H, Casas A, Chueke F, Paredes S and Batlle A (1993) Photodynamic action of endogenously syntesized porphyrins from aminolevulinic acid, using a new model for assaying the effectiveness of tumoral cell killing. *Int J Biochem* **25**: 1395–1398

Galli S, Colombo L, Vanzuli S, Daroqui M, Vidal M, Jasnis A, Lustig E and Eiján A (2000) Characterization of a fibroblastoid mammary carcinoma cell line (LM2) originated from a mouse adenocarcinoma. *Int J Oncol* **17**: 1259–1265

Gaullier J, Berg C, Peng Q, Anholt H, Selbo P, Ma L and Moan J (1997) Use of 5-aminolevulinic acid esters to improve photodynamic therapy on cells in culture. *Cancer Res* **57**: 1481–1486

Gossner L, Stolte M, Sroka R, Rick K, May A, Hahn E and Ell C (1998) Photodynamic ablation of high-grade dysplasia and early cancer in Barrett's esophagus by means of 5-aminolevulinic acid. *Gastroenterology* **114**: 448–455

He J, Whitacre C, Xue L, Berger N and Oleinick N (1998) Protease activation and cleavage of poly (ADP-ribose) polymerase: an integral part of apoptosis in response to photodynamic treatment. *Cancer Res* **58**: 940–946

He X, Sikes R, Thomsen S, Chung L and Jacques S (1994) Photodynamic therapy with Photofrin II induces programmed cell death in carcinoma cell lines. *Photochem Photobiol* **59**: 468–473

Jichlinski P, Forrer M, Mizeret J, Glanzmann T, Braichotte D, van den Bergh H and Leisinger H (1997) Clinical evaluation of a method for detecting superficial transitional cell carcinoma of the bladder by light-iduced fluorescence of protoporphyrin IX following topical application of 5-aminolevulinic acid: preliminary results. *Lasers Surg Med* **20**: 402–408

Kennedy J, Pottier R and Pross G (1990) Photodynamic Therapy with endogenous protoporphyrin IX: basic principles and present clinical experience. *J Photochem Photobiol B* **6**: 143–148

Kloek J and Beijersbergen van Henegouwen G (1996) Prodrugs for 5-Aminolevulinic acid for Photodynamic therapy. *Photochem Photobiol* **64**: 994–1000

Kloek J, Akkermans W and Beijersbergen van Henegouwen G (1998) Derivatives of 5-Aminolevulinic acid for Photodynamic therapy: enzymatic conversion into protoporphyrin. *Photochem Photobiol* **67**: 150–154

Kriegmair M, Baumgartner R, Knuechel R, Stepp H, Hofstädter F and Hofstetter A (1996) Detection of early bladder cancer by 5-aminolevulinic acid induced porphyrin fluorescence. *J Urology* **155**: 105–110

Lange N, Jichlinski P, Zellweger M, Forrer M, Marti A, Guillou L, Kucera P, Wagnieres G and van den Bergh H (1996) Photodetection of early human bladder cancer based on the fluorescence of 5-aminolevulinic acid hexylester-induced protoporphyrin IX: a pilot study. *Br J Cancer* **80**: 185–193

Lui H and Anderson R (1992) Photodynamic Therapy for the treatment of basal cell carcinoma. *Arch Dermatol* **128**: 1597–1601

Miyamoto Y, Umebayashi Y and Nishisaka T (1999) Comparison of phototoxicity mechanism between pulsed and continuous wave irradiation in photodynamic therapy. *J Photochem Photobiol B* **53**: 53–59

Moan J and Berg K (1992) Photochemotherapy of cancer: experimental research. *Photochem Photobiol* **55**: 931–948

Moan J, Bech O, Gaullier J, Stokke T, Steen H, Ma L and Berg K (1998) Protoporphyrin IX accumulation in cells treated with 5-aminolevulinic acid: dependence on cell density, cell size and cell cycle. *Int J Cancer* **75**: 134–139

Noodt B, Berg K, Stoke T, Peng Q and Nesland J (1996) Apoptosis and necrosis induced with light and 5-aminolaevulinic acid-derived protoporphyrin IX. *Br J Cancer* **74**: 22–29

Oseroff A. Photodynamic Therapy (1993) In *Clinical Photomedicine* (Edited by H. Lim and N. Sorter) pp. 387–402. Marcel Dekker, New York

Peng Q, Warloe T, Moan J, Heyerdahl H, Steen H, Giercksky K and Nesland J (1995) ALA derivative-induced protoporphyrin IX build-up and distribution in human nodular basal cell carcinoma. *Photochem Photobiol* **61**: Abstract 82S

Peng Q, Moan J, Warloe T, lani V, Steen H, Bjørseth A and Nesland J (1996) Build-up of esterified aminolevulinic-acid-derivative-induced porphyrin fluorescence in normal mouse skin. *J Photochem Photobiol B* **34**: 95–96

Rud E, Gederaas G, Brown S, Holroyd J, Vernon D, Hogset A, Moan J and Berg K (1999) Cellular uptake mechanisms for 5-aminolevulinic acid and 5-aminolevulinic methyl ester. 8th Congress Eur. Soc. Photobiol., Granada, Spain, Book of Abstracts, S136, pp. 85

Steinbach P, Weingandt H, Baumgartner R, Kriegmair M, Hofstadter F and Knuchel R (1995) Cellular fluorescence of the endogenous photosensitizer protoporphyrin IX following exposure to 5-aminolevulinic acid. *Photochem Photobiol* **62**: 887–895

Stummer W, Stocker S, Wagner S, Stepp H, Fritsch C, Goetz C, Goetz A, Kiefmann R and Reulen H (1998) Intraoperative detection of malignant glioma by 5-ALA-induced porphyrin fluorescence. *Neurosurgery* **42**: 518–525

Uehlinger P, Zellweger M, Wagnieres G, Juillerat-Jeanneret L, van den Bergh H and Lange N (2000) 5-aminolevulinic acid and its derivatives: physical chemical properties and protoporphyrin IX formation in cultured cells. *J Photochem Photobiol B* **54**: 72–80

Washbrook R and Riley P (1997) Comparison of 5-aminolaevulinic acid and its methyl ester as an inducer of porphyrin synthesis in cultured cells. *Br J Cancer* **75**: 1417–1420

Webber J, Luo Y, Crilly R, Fromm D and Kessel D (1996) An apoptotic response to photodynamic therapy with endogenous protoporphyrin *in vivo. J Photochem Photobiol B* **35**: 209–211

Wyss P, Fehr M, van den bergh H and Haller U (1998) Feasibility of photodynamic endometrial ablation without anesthesia. *Int J Gynec Obstet* **60**: 287–288

 © 2001 Cancer Research Campaign

## References

[PDF_00559] Agarwal M. L., Larkin H. E., Zaidi S. I., Mukhtar H., Oleinick N. L. (1993). Phospholipase activation triggers apoptosis in photosensitized mouse lymphoma cells.. Cancer Res.

[PDF_00562] Baumgartner R., Huber R. M., Schulz H., Stepp H., Rick K., Gamarra F., Leberig A., Roth C. (1996). Inhalation of 5-aminolevulinic acid: a new technique for fluorescence detection of early stage lung cancer.. J Photochem Photobiol B.

[PDF_00566] Casas A., Batlle A. M., Butler A. R., Robertson D., Brown E. H., MacRobert A., Riley P. A. (1999). Comparative effect of ALA derivatives on protoporphyrin IX production in human and rat skin organ cultures.. Br J Cancer.

[PDF_00573] Dellinger M. (1996). Apoptosis or necrosis following Photofrin photosensitization: influence of the incubation protocol.. Photochem Photobiol.

[PDF_00575] Denizot F., Lang R. (1986). Rapid colorimetric assay for cell growth and survival. Modifications to the tetrazolium dye procedure giving improved sensitivity and reliability.. J Immunol Methods.

[PDF_00578] Fan K. F., Hopper C., Speight P. M., Buonaccorsi G., MacRobert A. J., Bown S. G. (1996). Photodynamic therapy using 5-aminolevulinic acid for premalignant and malignant lesions of the oral cavity.. Cancer.

[PDF_00581] Fritsch C., Homey B., Stahl W., Lehmann P., Ruzicka T., Sies H. (1998). Preferential relative porphyrin enrichment in solar keratoses upon topical application of delta-aminolevulinic acid methylester.. Photochem Photobiol.

[PDF_00584] Fukuda H., Casas A., Chueke F., Paredes S., Batlle A. M. (1993). Photodynamic action of endogenously synthesized porphyrins from aminolevulinic acid, using a new model for assaying the effectiveness of tumoral cell killing.. Int J Biochem.

[PDF_00588] Galli S., Colombo L., Vanzuli S., Daroqui M. C., del Carmen Vidal M., Jasnis M. A., Sacerdote de Lustig E., Eijan A. M. (2000). Characterization of a fibroblastoid mammary carcinoma cell line (LM2) originated from a mouse adenocarcinoma.. Int J Oncol.

[PDF_00591] Gaullier J. M., Berg K., Peng Q., Anholt H., Selbo P. K., Ma L. W., Moan J. (1997). Use of 5-aminolevulinic acid esters to improve photodynamic therapy on cells in culture.. Cancer Res.

[PDF_00594] Gossner L., Stolte M., Sroka R., Rick K., May A., Hahn E. G., Ell C. (1998). Photodynamic ablation of high-grade dysplasia and early cancer in Barrett's esophagus by means of 5-aminolevulinic acid.. Gastroenterology.

[PDF_00598] He J., Whitacre C. M., Xue L. Y., Berger N. A., Oleinick N. L. (1998). Protease activation and cleavage of poly(ADP-ribose) polymerase: an integral part of apoptosis in response to photodynamic treatment.. Cancer Res.

[PDF_00601] He X. Y., Sikes R. A., Thomsen S., Chung L. W., Jacques S. L. (1994). Photodynamic therapy with photofrin II induces programmed cell death in carcinoma cell lines.. Photochem Photobiol.

[PDF_00604] Jichlinski P., Forrer M., Mizeret J., Glanzmann T., Braichotte D., Wagnières G., Zimmer G., Guillou L., Schmidlin F., Graber P. (1997). Clinical evaluation of a method for detecting superficial surgical transitional cell carcinoma of the bladder by light-induced fluorescence of protoporphyrin IX following the topical application of 5-aminolevulinic acid: preliminary results.. Lasers Surg Med.

[PDF_00609] Kennedy J. C., Pottier R. H., Pross D. C. (1990). Photodynamic therapy with endogenous protoporphyrin IX: basic principles and present clinical experience.. J Photochem Photobiol B.

[PDF_00615] Kloek J., Akkermans W., Beijersbergen van Henegouwen G. M. (1998). Derivatives of 5-aminolevulinic acid for photodynamic therapy: enzymatic conversion into protoporphyrin.. Photochem Photobiol.

[PDF_00612] Kloek J., Beijersbergen van Henegouwen (1996). Prodrugs of 5-aminolevulinic acid for photodynamic therapy.. Photochem Photobiol.

[PDF_00618] Kriegmair M., Baumgartner R., Knüchel R., Stepp H., Hofstädter F., Hofstetter A. (1996). Detection of early bladder cancer by 5-aminolevulinic acid induced porphyrin fluorescence.. J Urol.

[PDF_00621] Lange N., Jichlinski P., Zellweger M., Forrer M., Marti A., Guillou L., Kucera P., Wagnières G., van den Bergh H. (1999). Photodetection of early human bladder cancer based on the fluorescence of 5-aminolaevulinic acid hexylester-induced protoporphyrin IX: a pilot study.. Br J Cancer.

[PDF_00628] Miyamoto Y., Umebayashi Y., Nishisaka T. (1999). Comparison of phototoxicity mechanism between pulsed and continuous wave irradiation in photodynamic therapy.. J Photochem Photobiol B.

[PDF_00633] Moan J., Bech O., Gaullier J. M., Stokke T., Steen H. B., Ma L. W., Berg K. (1998). Protoporphyrin IX accumulation in cells treated with 5-aminolevulinic acid: dependence on cell density, cell size and cell cycle.. Int J Cancer.

[PDF_00631] Moan J., Berg K. (1992). Photochemotherapy of cancer: experimental research.. Photochem Photobiol.

[PDF_00636] Noodt B. B., Berg K., Stokke T., Peng Q., Nesland J. M. (1996). Apoptosis and necrosis induced with light and 5-aminolaevulinic acid-derived protoporphyrin IX.. Br J Cancer.

[PDF_00644] Peng Q., Moan J., Warloe T., Iani V., Steen H. B., Bjørseth A., Nesland J. M. (1996). Build-up of esterified aminolevulinic-acid-derivative-induced porphyrin fluorescence in normal mouse skin.. J Photochem Photobiol B.

[PDF_00570] Savin J. A. (2000). Osler and the skin.. Br J Dermatol.

[PDF_00651] Steinbach P., Weingandt H., Baumgartner R., Kriegmair M., Hofstädter F., Knüchel R. (1995). Cellular fluorescence of the endogenous photosensitizer protoporphyrin IX following exposure to 5-aminolevulinic acid.. Photochem Photobiol.

[PDF_00655] Stummer W., Stocker S., Wagner S., Stepp H., Fritsch C., Goetz C., Goetz A. E., Kiefmann R., Reulen H. J. (1998). Intraoperative detection of malignant gliomas by 5-aminolevulinic acid-induced porphyrin fluorescence.. Neurosurgery.

[PDF_00658] Uehlinger P., Zellweger M., Wagnières G., Juillerat-Jeanneret L., van den Bergh H., Lange N. (2000). 5-Aminolevulinic acid and its derivatives: physical chemical properties and protoporphyrin IX formation in cultured cells.. J Photochem Photobiol B.

[PDF_00662] Washbrook R., Riley P. A. (1997). Comparison of delta-aminolaevulinic acid and its methyl ester as an inducer of porphyrin synthesis in cultured cells.. Br J Cancer.

[PDF_00665] Webber J., Luo Y., Crilly R., Fromm D., Kessel D. (1996). An apoptotic response to photodynamic therapy with endogenous protoporphyrin in vivo.. J Photochem Photobiol B.

[PDF_00626] Wilson B. D., Mang T. S., Stoll H., Jones C., Cooper M., Dougherty T. J. (1992). Photodynamic therapy for the treatment of basal cell carcinoma.. Arch Dermatol.

[PDF_00668] Wyss P., Fehr M., Van den Bergh H., Haller U. (1998). Feasibility of photodynamic endometrial ablation without anesthesia.. Int J Gynaecol Obstet.

